# A multi modal approach to microstructure evolution and mechanical response of additive friction stir deposited AZ31B Mg alloy

**DOI:** 10.1038/s41598-022-17566-5

**Published:** 2022-08-02

**Authors:** Sameehan S. Joshi, Shashank Sharma, M. Radhakrishnan, Mangesh V. Pantawane, Shreyash M. Patil, Yuqi Jin, Teng Yang, Daniel A. Riley, Rajarshi Banerjee, Narendra B. Dahotre

**Affiliations:** 1 Department of Materials Science and Engineering, University of North Texas, 3940 N Elm St, Denton, TX 76207 USA; 2 Center for Agile and Adaptive Additive Manufacturing, University of North Texas, 3940 N Elm St, Denton, TX 76207 USA

**Keywords:** Mechanical properties, Metals and alloys

## Abstract

Current work explored solid-state additive manufacturing of AZ31B-Mg alloy using additive friction stir deposition. Samples with relative densities ≥ 99.4% were additively produced. Spatial and temporal evolution of temperature during additive friction stir deposition was predicted using multi-layer computational process model. Microstructural evolution in the additively fabricated samples was examined using electron back scatter diffraction and high-resolution transmission electron microscopy. Mechanical properties of the additive samples were evaluated by non-destructive effective bulk modulus elastography and destructive uni-axial tensile testing. Additively produced samples experienced evolution of predominantly basal texture on the top surface and a marginal increase in the grain size compared to feed stock. Transmission electron microscopy shed light on fine scale precipitation of Mg$_{17}$Al$_{12}$ within feed stock and additive samples. The fraction of Mg$_{17}$Al$_{12}$ reduced in the additively produced samples compared to feed stock. The bulk dynamic modulus of the additive samples was slightly lower than the feed stock. There was a $\sim\,$ 30 MPa reduction in 0.2% proof stress and a 10–30 MPa reduction in ultimate tensile strength for the additively produced samples compared to feed stock. The elongation of the additive samples was 4–10% lower than feed stock. Such a property response for additive friction stir deposited AZ31B-Mg alloy was realized through distinct thermokinetics driven multi-scale microstructure evolution.

## Introduction

Magnesium alloys find applications in automobile, aerospace, and biomedical industries due to high specific strength resulting from a low density of these materials^1–5^. Mg alloys also have excellent bio-compatibility^6,7^ and electromagnetic shielding capability^8^. However, Mg alloys have tendency to oxidize during casting and they develop strong texture during deformation, thus putting limitations on processing of Mg alloys using conventional methods such as casting and cold working^4,9^. Therefore, researchers explored strategies to overcome these limitations by using additive manufacturing (AM) routes such as laser beam additive manufacturing (LBAM), wire arc additive manufacturing (WAAM), and additive friction stir deposition (AFSD)^10–12^. LBAM and WAAM techniques are based on fusion of the feed material which is in the form of powder or wire. Both LBAM and WAAM techniques depend on melting and consolidation of the precursor material. On the other hand, AFSD is a solid state method. The feed material used during AFSD is in the form of rods or chips that are available commercially avoiding the usage of powder^13^. This is especially important for Mg as its powder is highly pyrophoric^14^.

AFSD works on the principle similar to friction stir processing (FSP). However, instead of a solid tool utilized for FSP, a hollow non-consumable tool is employed during AFSD. The feed material is fed through the hollow rotating tool which deforms plastically due to frictional heat generated between the tool, feed material, and the substrate. Such a friction results in softening of the feed material followed by its extrusion underneath the tool. The tool is then traversed for subsequent deposition of a layer. AFSD has evolved recently with development of AM machines such as MELD^®^. It has the ability of producing fully dense large components with complex geometries^15,16^. AM of conventional ferrous^17^ and non-ferrous^18–20^ alloys has been explored through AFSD.

Till date there have been very few reports published related to AFSD of Mg alloys^21–23^. Work by Calvert demonstrated successful deposition of WE43 Mg alloy through ASFD, but it lacked in explaining the evolution of microstructures in correlation to the process attributes^21^. Robinson et. al. demonstrated AFSD of AZ31B-Mg and examined the microstructural as well as mechanical property evolution^22^. The tensile test results showed that there was $\sim$ 20% drop in 0.2% proof stress (0.2% PS) and identical ultimate tensile strength (UTS) for the AFSD processed AZ31B-Mg compared to the wrought AZ31B-Mg material. This work provided a limited explanation and rationale behind such a lowering of the mechanical properties. In another effort, Williams et. al. deposited WE43 Mg alloy through AFSD^23^. Although these authors reported a $\sim$ 22 times reduction in grain size for the AFSD fabricated material compared to the feed stock, they still observed a $\sim$ 80 MPa reduction in 0.2% PS, $\sim$ 100 MPa reduction in UTS, and 11% reduction in elongation compared to the feed material. Whilst this work examined various processing conditions during AFSD, it lacked in physical explanation about the structure-property evolution in AFSD WE43 Mg alloy.

Based on above discussion, the mechanisms behind process-structure-property response in AFSD produced Mg alloys are not fully explored. Furthermore, compared to conventional FSP, AFSD involves addition of multiple layers which may result in subjecting the previously deposited material to repetitive thermokinetics thereby potentially impacting the microstructure evolution. Experimental monitoring of thermophysical parameters during such a complex process is difficult and limited in terms of spatial as well as temporal resolution. In light of this, computational modeling of the multi layer additive deposition process can provide insights into the thermokinetic effects experienced by the AFSD produced material throughout the process. Such predictions of thermokinetics could be vital in uncovering the processing-structure-property response in the AFSD fabricated material. While there are multiple computational modeling efforts related to conventional FSP and rotary friction welding (RFW)^24–27^, there is sparsity of literature related to simulation of AFSD process. Recently, a smooth particle hydrodynamics-based AFSD model has been proposed^28^. However, the model was restricted to a single deposition track, thus lacking in prediction of the effects of repetitive thermokinetics associated with subsequently added layers. Furthermore, the reported computational run time was substantially high (> 30 hrs).

In light of the limited experimental and computational efforts related to the AFSD process highlighted above, the current work systematically investigated the multi scale microstructure evolution and resultant mechanical property response in AFSD AZ31B-Mg alloy. The microstructure observations were explained using spatial and temporal thermokinetics predicted by a multi layer computational process model. The mechanical properties of the AFSD AZ31B-Mg were evaluated using non destructive effective bulk modulus elastography (EBME) and destructive uni-axial tensile tests. The observed property response was analyzed based on the micro and nano scale structural changes experienced by the AFSD processed material compared to the feed stock. The current work formed as a part of continuation of efforts by the present research group focusing on the advanced processing of the Mg alloys^2,6,29–37^.

## Methods and materials

### Additive friction stir deposition

**Figure 1:**
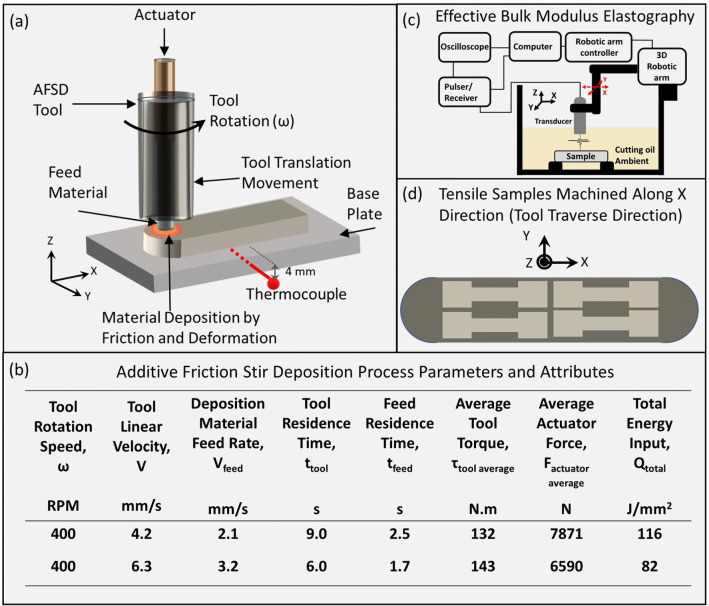
Schematics of (**a**) the AFSD process, (**b**) important AFSD process parameters and attributes employed in the current work,  (**c**) non destructive testing via EBME Method, and (**d**) location of tensile specimen machined along tool traverse direction through the thickness of the AFSD deposits.

AFSD fabrication was conducted on MELD^®^ machine equipped with a hollow cylindrical tool containing coaxial cavity of 9.$5 \times 9$.5 mm$^2$ cross-section (Fig. 1a). Outer diameter and height of the AFSD tool were 38.1 mm and 138 mm respectively. Commercially available AZ31B-Mg (chemical composition in wt%: Mg-3w%Al-1%Zn-0.5%Mn) bar stock in H24 temper condition with dimensions 9.$5 \times 9$.$5 \times 460$ mm$^3$ was fed into the actuator setup through the hollow AFSD tool. The H24 temper treatment for the feed material consisted of forming the material below 160 °C followed by annealing in the temperature range of 150–180 °C^38^. AZ31B-Mg plate was utilized as the substrate plate during AFSD. It is worth noting here that, the current study formed as a continuation effort of the previous publication by the authors related to the process optimization aspects of the AFSD fabrication of AZ31B Mg alloy^37^. Several preliminary trials were conducted to carry out AFSD of AZ31B Mg material to select the AFSD process parameters leading to successful AFSD fabrication of AZ31B Mg. The tool rotation velocity was maintained at 400 rpm, whereas, the tool linear velocities of 4.2 and 6.3 mm/s were implemented in the AFSD processing during the current work. It was observed during initial multiple trials that the successful deposition with minimal flash occurred when the feed rate for the bar stock was maintained at $\sim$ 50% of the tool linear velocity. A layer of material was deposited with 140 mm length and the tool was shifted upwards by 1 mm to deposit a subsequent layer. A total of 5 layers were deposited with each set of processing condition. The onboard sensors monitored variation in tool torque and actuator force as a function of time during each AFSD condition. A type K thermocouple was embedded 4 mm below surface of substrate plate at a location directly below the center of AFSD deposit to monitor the temporal variation of temperature during deposition.

The tool residence time (t$_{tool}$) and feed residence time (t$_{feed}$) were estimated using following equations

1 $$\begin{aligned} t_{tool} = \frac{2\times R_{tool}}{V_{linear}} \end{aligned}$$

where R$_{tool}$ is the outer radius of the tool and V$_{linear}$ is the tool linear velocity

2 $$\begin{aligned} t_{feed} = \frac{2\times R_{feed}}{V_{linear}} \end{aligned}$$

where R$_{feed}$ is the equivalent circular radius of the feed material (5.3 mm).

The heat input imparted by the tool (H$_{tool}$) due to tool torque was expressed as^39,40^

3 $$\begin{aligned} H_{tool} = \frac{4\pi ^2}{3} \omega \left( \frac{\tau _{tool\;average} }{R_{tool}(A_{tool}-A_{feed})}\right) \; (R^3_{tool}-R^3_{feed}) \end{aligned}$$

where $\omega$ is the rotational velocity of tool-feed assembly, $\tau _{tool\;average}$ is the average torque experienced by the AFSD tool during the deposition (Fig. 1 b), A$_{tool}$ is the area of cross-section of the tool, and A$_{feed}$ is the area of cross-section of the feed. Similarly, the heat input corresponding to the feed stock (H$_{feed}$) was derived as follows^39,40^

4 $$\begin{aligned} H_{feed} = \frac{4\pi ^2}{3}\mu \omega \left( \frac{F_{actuator\;average}}{A_{feed}}\right) \; (R^3_{feed}-3R^2_{feed}.h) \end{aligned}$$

where $\mu$ is the coefficient of friction (0.6) between feed stock and the base plate^41^, F$_{actuator\;average}$ is the average actuator force acting upon feed material during deposition (Fig. 1 b), and h is the layer thickness.

Finally, the total energy input per unit area Q$_{total}$ was estimated as

5 $$\begin{aligned} Q_{total} = Q_{tool}+ Q_{feed} = \frac{H_{tool}.t_{tool}}{A_{tool}} + \frac{H_{feed}.t_{feed}}{A_{feed}} \end{aligned}$$

where Q$_{feed}$ and Q$_{feed}$ are energy inputs per unit area for tool and feed stock respectively. The process parameters, values of average tool torque, average actuator force, and computed total energy inputs are presented in Fig. 1b. Further details about the computations of heat and energy inputs during AFSD process can be located in previous publication by the present research group^37^.

### Examination of multi-scale microstructure

As an initial step, the as fabricated samples were visually observed and then sectioned for successive analysis. Density of the sectioned samples was evaluated using Archimedes method with the aid of a high precision Sartorius micro-balance based on the protocol provided in ASTM B962 standard^42^. At least 3 samples were evaluated for density for each AFSD processing condition. Microstructural characterization of the as-received feed stock and AFSD processed AZ31B-Mg samples was performed in X-Z plane by electron back-scattered diffraction (EBSD) in a scanning electron microscope (SEM) and transmission electron microscopy (TEM) techniques. The samples were sectioned from the central steady state zone. Samples for EBSD were prepared with preliminary mechanical polishing employing SiC papers in the range of 800–1200 grit with ethanol as a lubricant. The samples were then transferred to Buehler textmet cloths containing diamond suspensions with average particle sizes of 1 and 0.25 μm respectively to obtain a mirror finished surface. The mechanically polished AZ31B-Mg samples appeared to develop the oxide layer, which prevented obtaining Kikuchi signals during EBSD. This issue was addressed by ion polishing using a Gatan 682 precision etching coating system with the ion beam current of 190 μA and voltage of 5 keV. The sample surface was inclined at 4° with respect to the ion beam and polished for 30 s. EBSD was performed using a Thermo-Fisher Nova NanoSEM 230 operating at 20 keV equipped with a Hikari super EBSD detector. The sample surface was tilted with respect to the primary electron beam by mounting on 70° pre-tilted holder kept at a working distance of 12 mm. The generated data were further analyzed in TSL OIM analysis 8.0 software, where orientation image maps (OIM) and pole figures were generated. To represent the micro-texture on normal plane of the processed samples, measured data in the X-Z plane of the AFSD sample were rotated by 90° around X-axis. A similar approach was adopted for the feed stock material. For better statistics and data consistency of grain sizes and micro-texture, multiple OIM scans (5) were taken from each sample condition.

Cross-sectional TEM foils were prepared using a Thermo-Fisher Nova 200 Nanolab dual beam focused ion beam (FIB) microscope. A 30 KV Ga$^{2+}$ beam was used in making trenches and for initial thinning of the foils. Final thinning to foil thickness less than 100 nm was made with a 5 keV Ga$^{2+}$ beam. A platinum coating was deposited to protect the processed sample surface from ion beam damage. TEM imaging was performed using a Thermo-Fisher Tecnai G2 F20 microscope operating at 200 keV to obtain both bright field and dark field micrographs along with corresponding selected area diffraction patterns (SADP).

### Mechanical evaluation

As a first level of mechanical property evaluation, dynamic elastic constants of the feed stock and AFSD samples were measured using the non-destructive EBME method (Fig. 1c). These tests were performed inside a 480 $\hbox {mm} \times 300$ $\hbox {mm} \times 180$ mm glass tank filled with commercially available cutting oil, where the sample and longitudinal transducer were completely immersed, as depicted in Fig. 1c. An Olympus V211 0.125-inch diameter 20 MHz planar immersion-style transducer was used to excite a broadband pulse from 13 to 27 MHz with a repetition rate of 2 ms. The scanning motion was accurately controlled by the UR5 robotic arm using MATLAB script. A JSR Ultrasonic DPR 500 Pulse/ Receiver provided the pulse source and time trigger, and the data was collected by a Tektronix MDO 304 at 1 GHz sampling rate. The contours were raster-scanned with the areas of 100 mm$\times$25 mm with 1 mm spatial intervals. At each scanned location, the scan was paused for 20 s for collecting the average of the 512 acoustic signals. The transducer surface aligned parallel to the sample surface (XY plane) with a distance of more than 2 wavelengths. In present experiments, the recorded signals were the reflections from the upper and lower sample surfaces. The additional fundamental details of the EBME process employed to obtain the dynamic elastic constants are provided in the earlier reports of the authors^43,44^.

Next level of mechanical evaluation of AZ31B-Mg feed stock and AFSD samples was carried out using uni-axial tensile testing. Flat dog bone shaped tensile specimen with the gage length of 25 mm and thickness of 1.5 mm in accordance with ASTM E8 standard^45^ were machined out along the length of the deposited sample using wire electrical discharge machine (EDM) (Fig. 1d). The tensile tests were conducted as per ASTM E8 standard employing a strain rate of 10$^{-4}$/s on 25 kN load cell Instron universal testing machine equipped with an extensometer. At least 4 samples were tested for each AFSD condition and the feed material. Values of Young’s modulus, 0.2% PS, UTS, and % elongation were estimated from the recorded engineering stress-strain curves.

### Multi layer computational process model

**Figure 2:**
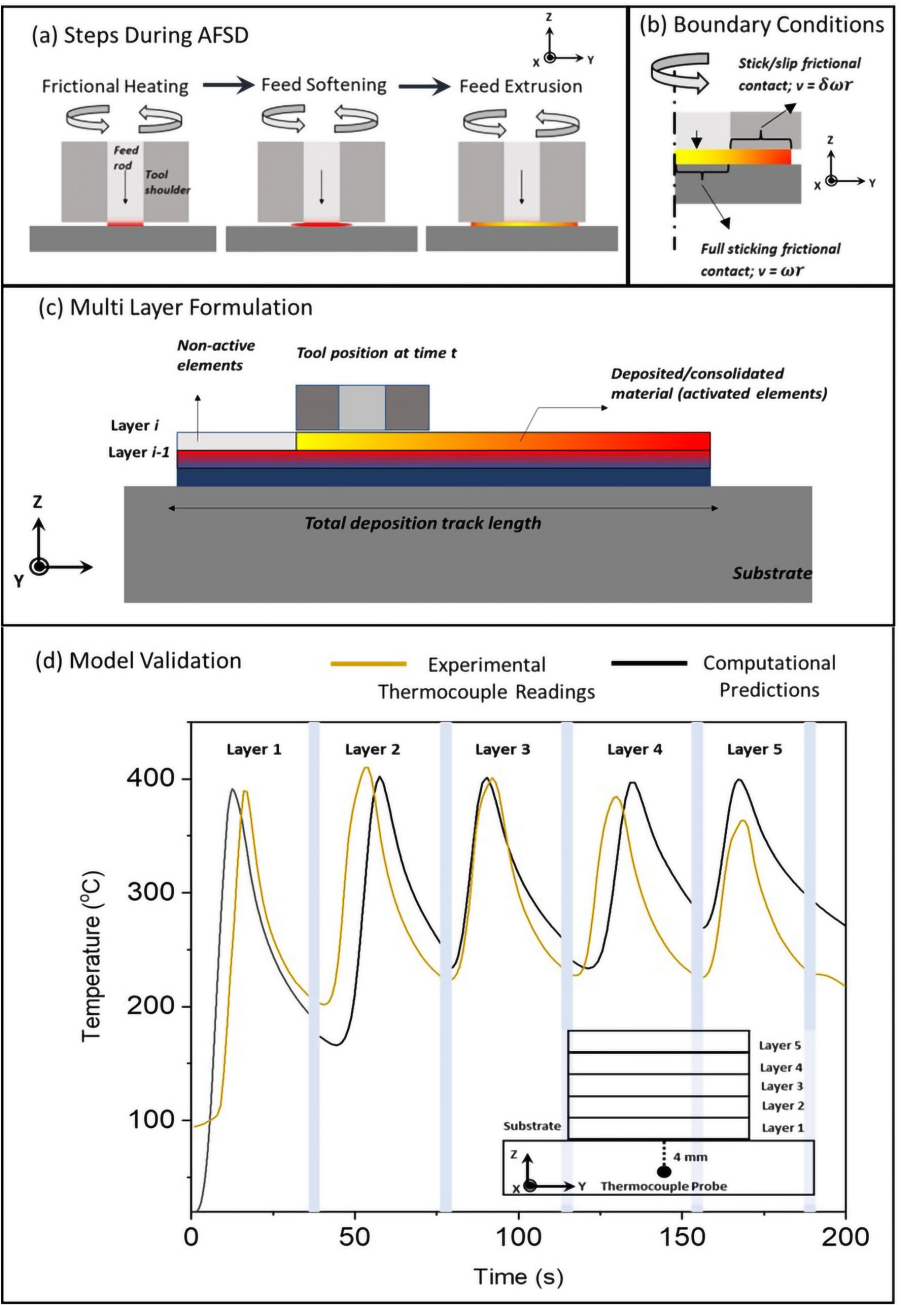
Schematics of multi-layer computational model methodology adopted in the current work showing (**a**) steps in AFSD process, (**b**) model boundary conditions, (**c**) multi layer formulation approach, and (**d**) validation exercise with the aid of time temperature plots showing thermocouple readings and multi layer computational process model predictions.

A computational model of multi-layer process was employed to predict the spatial and temporal variation in temperature during AFSD fabrication of AZ31B-Mg alloy. AFSD comprises of multiple unique phenomena such as feed rod deformation, material extrusion, stirring and deposition compared to other friction based processing techniques^18^. Sequential events of interactions among feed, tool, and substrate materials during AFSD as discussed in the “Introduction” section were taken into consideration while formulating the computational process model (Fig. 2a–c). These steps were repeated during simulation of total 5 layers. In AFSD, the primary source of heat generation can be attributed to frictional contact between feed rod/substrate interface and the extruded material/tool shoulder interface (Fig. 2). A multi-layer frictional heating thermal model for AFSD was developed employing the governing equation pertaining to conduction-based heat transfer as expressed below:

6 $$\begin{aligned} \rho C_p\ \frac{\partial T}{\partial t}+\rho C_p\left( {\vec {u}}\cdot \nabla T\right) =\nabla \cdot \left( k\nabla T\right) +{q_p}^{\prime \prime \prime } \end{aligned}$$

In the above equation, T is temperature, t is time, $\rho$ represents density (kg/m$^3$), C$_p$ is specific heat (kJ/mol), and ${\vec {u}}$ is advection velocity. Importantly, the term q$_p$′′′ represents volumetric heat generation. In context of AFSD q$_p$ can be related to volumetric heat generation due to plastic deformation. However, formulation of q$_p$′′′ requires detailed information about plastic strains rates and flow stress, which is computationally taxing (thermomechanical or CFD model is required) and challenging, especially for multi-layer modeling framework^25,46^. In light of this, only frictional heating during AFSD was considered in a surface heat flux boundary condition based on a simple theory of pure conduction models associated with friction stir welding (FSW)^24,47^. Thus, the boundary heat flux q$_f$ due to frictional contact between feed rod/substrate interface can be expressed as:

7 $$\begin{aligned} q_f=\tau _{yield}\times \left( \omega R-V_{linear}\ sin\theta \right) ;\ 0<R\le R_{feed} \end{aligned}$$

$\tau _{yield}$ corresponds to shear stress experienced by the deforming material at the feed rod/substrate interface and R is the distance from center of the feed towards the feed edge. The assumption underlying above formulation is based on the existence of a fully sticking contact at the interface under plastic deformation of the feed material (Fig. 2b). When the feed material thermally softens via plasticization, the shear stress $\tau _{yield}$ under sticking assumptions can be expressed as^48^

8 $$\begin{aligned} \tau _{yield}=\frac{\sigma _{yield}}{\sqrt{3}} \end{aligned}$$

where $\sigma _{yield}$ is the temperature dependent yield strength of the depositing material available for AZ31B-Mg alloy in the open literature^41^. Similarly, the surface heat flux at extruded material/tool shoulder interface (Fig. 2b) can be expressed as following

9 $$\begin{aligned} q_s=M_{tool\;average}\times (1-\delta )\times \left( \omega R-V_{linear}\ sin\theta \right) ;\ R_{feed}<R\le R_{tool} \end{aligned}$$

$\delta$ corresponds to slip-rate signifying the sliding/sticking contact state of the extruded material under tool shoulder. Thus, $\delta =0$ corresponds to fully sticking regime and $\delta =1$ denotes fully sliding regime. Thus, in case of sliding/sticking regime the value of $\delta$ ranges anywhere from 1 to 0. The term M$_{tool\;average}$ is derived from back calculation using experimentally obtained tool torque $\tau _{tool\;average}$ data during deposition^37^, as explained below

10 $$\begin{aligned} \tau _{tool\;average}=\int _{0}^{2\pi }\int _{R_{feed}}^{R_{tool}}{\eta \times r\times \left( M_{tool\;average}R\ dR\ d\theta \right) } \end{aligned}$$

where $\eta$ is mechanical efficiency. Furthermore, the slip rate^49^ can be expressed as

11 $$\begin{aligned} \delta =1-exp\left( \frac{-\delta _o\omega \left( R-R_{feed}\right) }{\omega _o\left( R_{tool}-R_{feed}\right) }\right) \end{aligned}$$

where $\delta _o$ is a scaling constant and $\omega _o$ is the reference value for the rotational tool speed. According to experimental observations, these values are adjusted to represent material flowability under the tool shoulder. For instance, for a given material that gets readily extruded, covering a large portion of the tool shoulder area, the term $(1-\delta )$ should gradually shift from 1 towards zero as *R* changes from $R_{feed}$ to $R_{tool}$ and vice versa. Thus, the above two position-dependent boundary heat flux conditions prescribe the thermal contribution in the developed model.

Figure 2b illustrates the schematic representation of the longitudinal cross-section of the computational domain. A quiet element activation/deactivation strategy was employed to incorporate the multi-layer deposition^50,51^. For any given point during deposition, the material preceding the moving tool area corresponds to deposited material. Hence, the material properties of the consolidated material were assigned to those elements. For the rest of the elements, material properties of air were assigned.

Lastly, all the boundaries associated with deposited material (contingent upon tool position and activation status) were assigned convective and radiative boundary conditions as expressed below:

12 $$\begin{aligned} q_{loss}=h_\infty (T_\infty -T)+\varepsilon \sigma (T_\infty ^4-T^4) \end{aligned}$$

where q$_{loss}$ is flux due to heat losses, h$_{\infty }$ is the convection coefficient, T$_{\infty }$ is the ambient temperature, $\epsilon$ is the emissivity, $\sigma$ is Stefan-Boltzmann constant. The thermophysical parameters discussed above are temperature dependent. The above mathematical model was executed on commercial FEA software COMSOL^®^ Multiphysics. An adaptive meshing strategy (dependent upon temperature and thermal gradient of mesh elements) were employed to achieve reasonable computational time considering the pure conduction problem. The dimension of each deposited track was 140$\times$38$\times$1 mm$^3$. Accordingly, the adaptive meshing strategy ensures a minimum element size of 1 mm in the thermally optimum region. The choice of 1 mm element size was based on mesh sensitivity analysis. The computational time for consecutive 5-layer deposition was less than 20 minutes on an Intel(R) Xeon (R) (Gold 6252 CPU @2.10 GHz–190 GB) processor.

The validation of the proposed thermal model was assessed using thermocouple temperature measurements. Figure 2d depicts the comparison between thermokinetic parameters (time and temperature) at any given locations within the AFSD layers measured by a thermocouple and predicted by a computational simulation. The temperature-time cycles in Fig. 2d are associated with the locations at the center of each AFSD layer corresponding to thermocouple based measurements and computational predictions. As can be observed, the thermal model provides reasonable agreement with the actual thermal evolution during the AFSD process. The minor variations from the actual temperature profile (Fig. 2d) can be attributed to heat generation due to plastic dissipation being neglected in the thermal model and smaller computational domain size compared to the experimentally used AZ31B-Mg base plate. Nevertheless, the proposed thermal model provides valuable information on layer-by-layer thermal evolution during AFSD. As a side note, a parallel study is underway in the current research group focusing on coupled thermal and thermomechancial phenomena during AFSD process and authors intend to report these results in a separate manuscript. Nonetheless, attempts were made to explain the microstructure evolution in correlation with the computationally predicted thermokinetic parameters in AFSD fabricated AZ31B-Mg.

## Results and discussion

The AFSD fabricated samples were  examined visually prior to cutting for microstructure observations. Although oxidation is a concern during additive fabrication of Mg based materials, and it is likely that there may be some oxygen pickup during AFSD of AZ31B Mg, no oxide layers were detected during visual observations of the AFSD fabricated samples. In general, AFSD being a solid-state process, the oxygen diffusion in solid is likely to be slow to introduce recognizable amount of oxygen in AZ31B Mg during processing. After visual observations, the samples were cut and prepared for successive set of observations. The Archimedes density of sectioned samples was measured as per ASTM B962 standard^42^. The average density values were $1.761 \pm 0.006$ and $1.768 \pm 0.006$ g/cm$^3$ for the samples corresponding to input energies of 82 and 116 J/mm$^2$ respectively as against the density value of 1.77 g/cm$^3$ for the feed stock material. This corresponds to relative density values of 99.4 and 99.8% for 82 and 116 J/mm$^2$ samples respectively indicating that a reasonable consolidation of material was achieved during AFSD process under the set of processing parameters employed in the present efforts.

**Figure 3:**
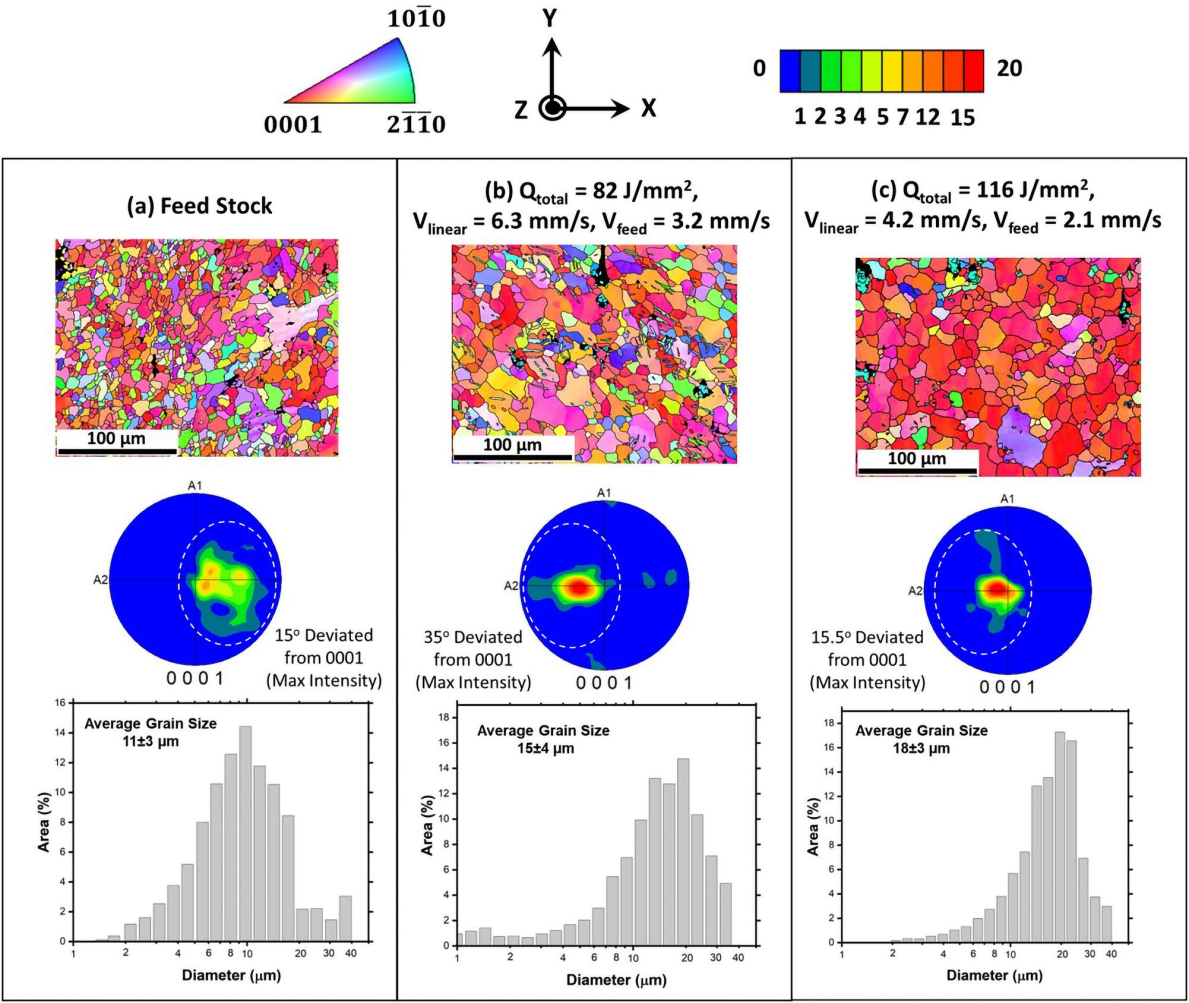
EBSD data showing OIM, texture plots, and grain size distributions corresponding to (**a**) feed stock, (**b**) 82 J/mm$^2$, and (**c**) 116 J/mm$^2$ samples.

First level of microscopy observations on AFSD AZ31B-Mg were performed using SEM-EBSD. OIM maps qualitatively indicated that the AFSD samples experienced a recognizable increase in the grain size compared to the feed stock (Fig. 3). This was also statistically confirmed from the grain size distribution, where the average grain size in both 82 J/mm$^2$ (15 ± 4 μm) and 116 J/mm$^2$ (18 ± 3 μm) AFSD samples was 1.4–1.6 fold higher compared to the feed stock (11 ± 3 μm) (Fig. 3). An increase in grain size after the AFSD process can occur due to dynamic recrystallization and grain growth mechanisms as the feed stock undergoes severe plastic deformation accompanied by the simultaneous generation and accumulation of heat during the AFSD process^52^.

In addition to grain size, the crystallographic texture evolution after the AFSD process can also be noticed in 0001 pole figures (Fig. 3). The crystallographic textures in all three samples were close to basal plane texture, and the texture appears to sharpen with an increase in the input energy from 82 J/mm$^2$ to 116 J/mm$^2$. The feed stock exhibited a substantially large spread ($\sim$ 30°) around the maximum texture intensity and the location of maximum intensity was 15° away from the ideal basal pole location (Fig. 3a). For 82 J/mm$^2$ and 116 J/mm$^2$ samples, the maximum texture intensities were observed to deviate 35 and 15.5° respectively from the basal pole, and the orientation spread was found to be $\sim$ 25° around the maximum intensity in both the cases (Fig. 3b, c).

**Figure 4:**
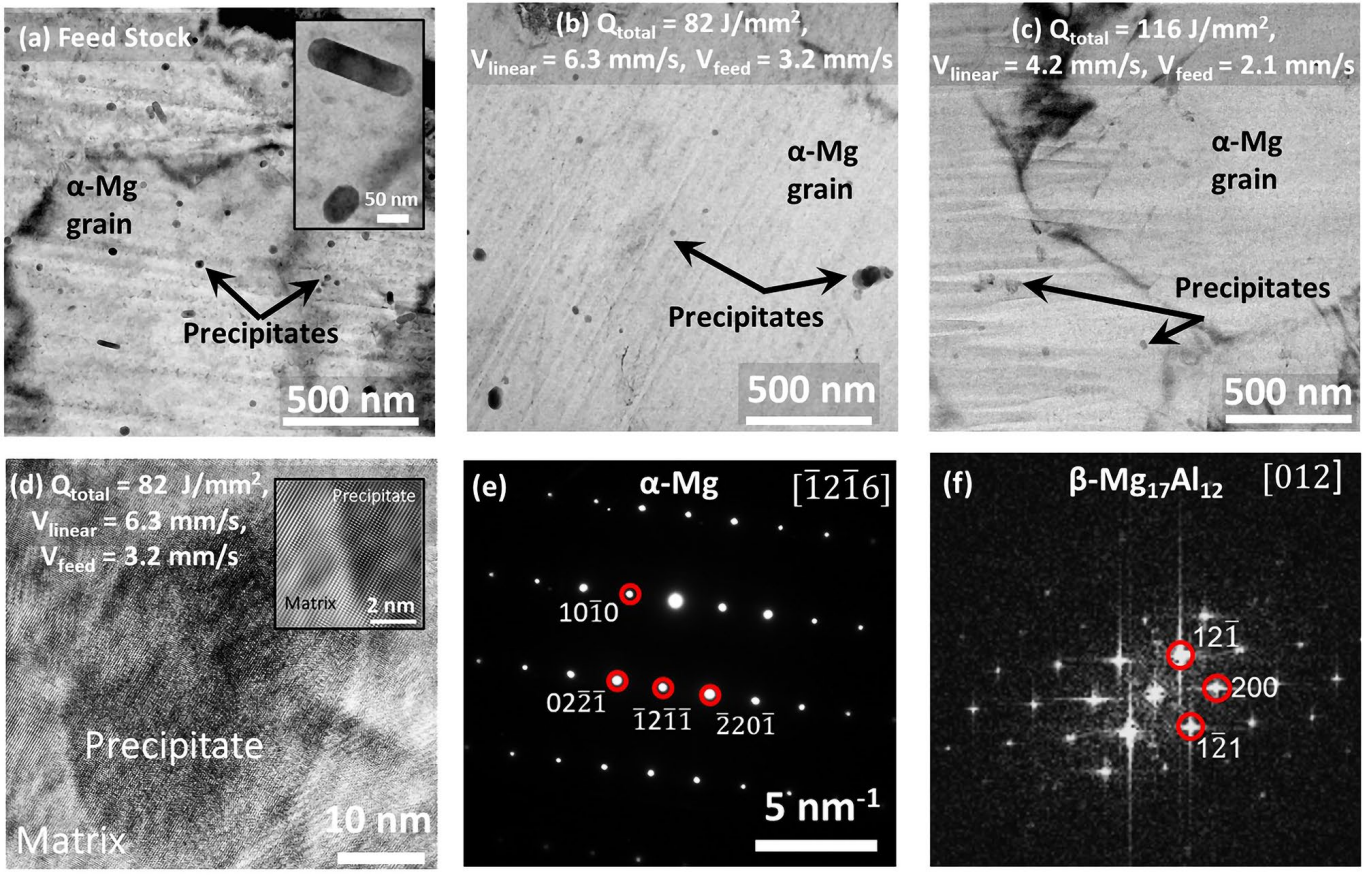
TEM data showing bright field images for (**a**) feed stock with inset showing magnified view of the precipitates, (**b**) 82 J/mm$^2$ AFSD sample, (**c**) 116 J/mm$^2$ AFSD sample, and (**d**) high resolution view of precipitate in 82 J/mm$^2$ AFSD sample with inset showing the coherent interface between precipitate and the matrix. The selected area diffraction pattern in (**e**) corresponds to  $\alpha$-Mg matrix and (**f**) is the fast Fourier transform image depicting $\beta$ Mg$_{17}$Al$_{12}$ precipitate.

To seek further insight into microstructure and phase evolutions, the AZ31B-Mg feed stock and AFSD samples were observed using high resolution TEM imaging (Fig. 4). The bright field (BF) TEM image corresponding to the feed stock revealed a uniform distribution of nm sized second phase precipitates (Fig. 4a). These precipitates exhibited both spherical and elongated morphologies in these TEM images. However, it should be noted that both morphologies are likely to be the same type of precipitate, viewed along two orthogonal directions. Therefore, it is likely that these precipitates have a cylindrical or cigar shaped morphology in three dimensions (inset of Fig. 4a). The sizes of the precipitates ranged between 20 and 60 nm. Although, the fraction of precipitates in both the AFSD samples was significantly reduced (Fig. 4b, c) compared to the feed stock (Fig. 4a), these second phase precipitates possessed an atomically coherent interface with the matrix (Fig. 4d). The SADP analysis revealed matrix as $\alpha$-Mg phase (Fig. 4e) while the second phase precipitates were $\beta$ Mg$_{17}$Al$_{12}$ phase as confirmed by the FFT pattern (Fig. 4f). In addition, no oxide phases were detected during high resolution TEM observations which was consistent with the visual observations noted before. A qualitative comparison of the microstructures suggests that with increasing deformation energy imposed during the AFSD processing, the fraction of precipitates significantly reduced. Additionally, the AFSD processed samples exhibited coarser grain size (Fig. 4b–c) as confirmed earlier through EBSD analysis (Fig. 3). In addition, the matrix grains of both the AFSD samples (82 and 116 J/mm$^2$) appeared to be free of dislocation contrast pointing towards possible restoration mechanisms (Fig. 4b–c). The process-induced dissolution of precipitates is attributed to the combination of spatial and temporal thermokinetic effects, which are discussed in the subsequent paragraphs.

**Figure 5:**
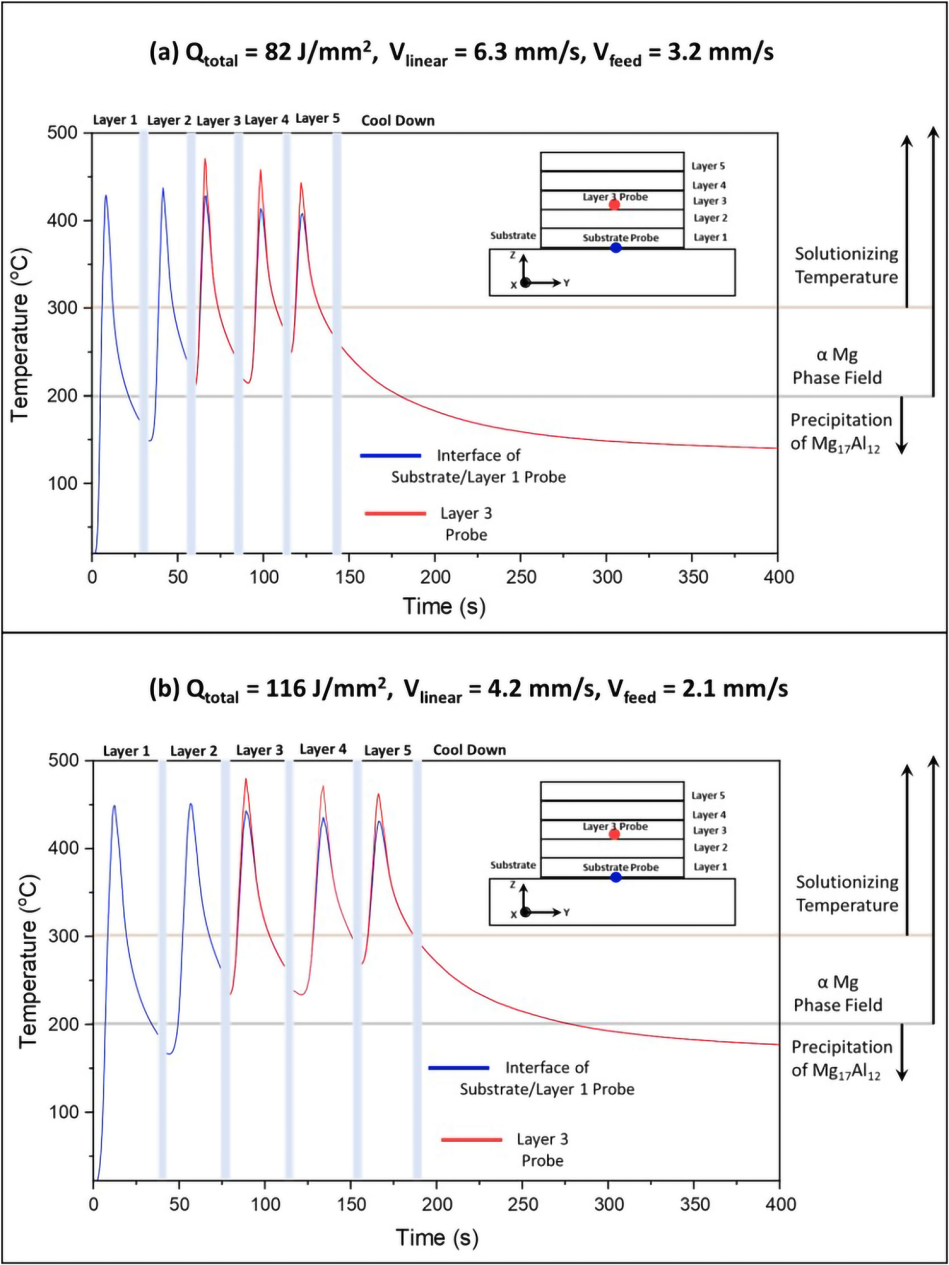
Predicted time-temperature plots for AFSD process using a multi layer computational process model corresponding to (**a**) 82 J/mm$^2$ and (**b**) 116 J/mm$^2$ samples. Important phase transition and conventional heat treatment temperature ranges are indicated for reference.

In order to realize the thermokinetic effects of AFSD process on the distinct microstructure evolution in processed AZ31B-Mg described above, the spatial and temporal variation of temperature during AFSD as predicted by the multi layer computational process model was examined (Fig. 5). The temperature was probed at the center of AFSD track at the interface between layer 1 and the substrate as well as at a location within layer 3 (100 μm above interfaces between layers 2 and 3). A virtual probe location at the interface of layer 1 and the substrate experienced a first single thermal cycle during fabrication of layer 1, where it achieved the maximum temperature of 430 °C for 82 J/mm$^2$ sample (Fig. 5a) and 450 °C for 116 J/mm$^2$ sample (Fig. 5b) at the instance of deposition. Subsequent thermal cycles (#s-2, 3, 4, and 5) were experienced by the probe location during the fabrication of successive layers (layer 2–5) resulting in the reheating of deposited material at the probe location in the corresponding preceding layers for both the AFSD conditions. The peak temperatures developed during deposition of subsequent layers were above 400 °C at any virtual location in layer 1 for both the AFSD conditions (Fig. 5). Notably, a slight increase ($\sim$ 5–10 °C) in the maximum temperature of the second reheating thermal cycle due to heat accumulation was observed in both the AFSD samples (Fig. 5a and b). The maximum temperature achieved at any virtual location in layer 1 due to subsequent reheating thermal cycles (corresponding to layers 3–5) decreased gradually in both the AFSD samples as a result of increasing distance between the probing location and the layer being deposited (Fig. 5a and b). The lowest temperature within layer 1 during reheating cycle while layer 2 was deposited on the top of it was above 150 °C and subsequent deposition of layers 3-5 reheated the material in layer 1 above 200 °C.

It is apparent that the probe location in layer 3 experienced thermal cycles only thrice during the fabrication of layers 3, 4, and 5, as predicted in Fig. 5. The heat accumulation effect is distinct from the maximum temperature of first thermal cycles experienced by location in layer 3 compared to that of the location lying at the interface of substrate and layer 1 for both AFSD conditions (Fig. 5). In addition, due to higher linear deposition velocity of 6.3 mm/s for 82 J/mm$^2$ compared to 4.2 mm/s for 116 J/mm$^2$, the average durations of corresponding thermal cycles were $\sim$ 30 and 38 s respectively (Fig. 5). Such distinct characteristics of heating-reheating cycles imposed on AFSD fabricated material influenced the microstructure evolution as described below.

According to the equilibrium Mg–Al phase diagram, above 200 °C, the $\beta$ phase (Mg$_{17}$Al$_{12}$) is thermodynamically unstable and undergoes dissolution to form a single-phase $\alpha$-Mg^53^. As discussed before, during the entire AFSD process, the reheating experienced by the previously deposited material kept the temperatures in the single $\alpha$-Mg phase regime at any virtual location within the previously deposited material (Fig. 5). The solutionizing temperatures for AZ31B-Mg have been reported to be in the range of 250–400 °C^54,55^. Upon conclusion of the AFSD process, the deposit cooled down to room temperature with the cooling rates in the range of 1–2 °C/s. However, the re-precipitation of $\beta$ phase may occur below the 200 °C provided there is no significant diffusion of Al away from the precipitate. In conventional processing, the aging of few hours is required to uniformly precipitate $\beta$ phase^56^. Based on the spatial and temporal thermal history predicted by the computational process model (Fig. 5), it was likely that the deposited AZ31B-Mg material remained in single $\alpha$-Mg phase field during the entire time of the AFSD process. To further quantitatively verify the dissolution of $\beta$ phase during deposition and possibility of re-precipitation of $\beta$ phase during cooling, extent of Al diffusion affected by process thermokinetics was computed for both the AFSD conditions. The computationally predicted thermal cycles, especially those corresponding to the location lying within the layer 3 were more relevant to understand the $\beta$ precipitate dissolution/re-precipitation as the microscopy observations were conducted in this region. Since the $\beta$ precipitate becomes thermodynamically unstable above 200 °C, precipitate dissolution occurs and aluminum atoms driven by local temperature rise can diffuse away from the precipitate site. The solution to Fick’s second law of diffusion with varying diffusion coefficients gives concentration spread with distance and time. Using its general solution, the diffusion length (x’) could be estimated over a period of time as follows:

13 $$\begin{aligned} x' = \sqrt{D(T).t} \end{aligned}$$

where D(T) is the diffusion coefficient as a function of temperature. The diffusion coefficient is expressed in Arrhenius form giving its temperature dependence as follows:

14 $$\begin{aligned} D(T) = D_{0} exp{\frac{-E}{RT}} \end{aligned}$$

where D$_0$ is the diffusion constant (3.275$\times$10$^-5$ m$^2$/s^57^), R is the gas constant (8.314 J/(mol K)), and E is the activation energy corresponding to stress-free lattice (E= 130.4$\times$10$^3$ J/mol^57^). However, E is also affected by the overall residual stress present in the material. The nature of stress present decides the resultant value of the activation energy. For instance, overall compressive stress would increase the activation energy while tensile stress would decrease it^58^. Accordingly, the following equation of diffusion coefficient dependent on temperature and stress was considered.

15 $$\begin{aligned} D(T) = D_{0} exp\left( \frac{-[E\pm (\frac{\sigma \times \Omega }{3})]}{RT}\right) \end{aligned}$$

where $\sigma$ is the stress (130 MPa as limiting experimentally observed value in the present case) and  $\Omega$ is the molar volume $(1.399 \times 10^{-5}$ m$^3$/mol). With the above equation, diffusion is primarily dependent on temperature. However, with the temperature-time relation obtained from the computational model (Fig. 5), diffusion coefficient dependence on time was obtained. This exercise allowed integration of Eq. 13 over a definite time range as follows:

16 $$\begin{aligned} x'^2 = \int _{t_1}^{t_2} D(T)dt \end{aligned}$$

**Figure 6:**
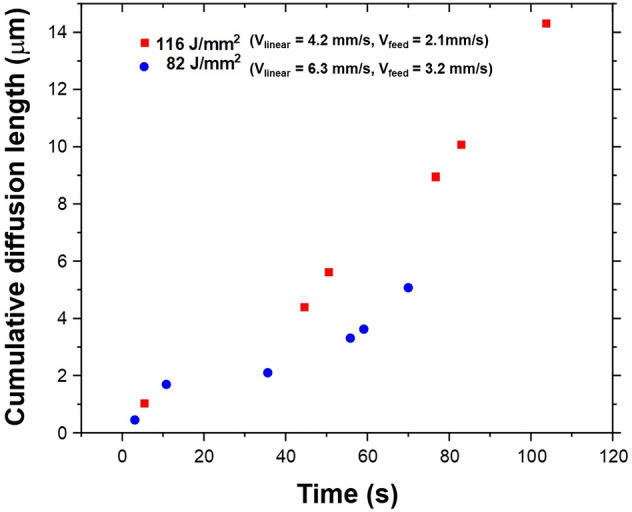
Computed cumulative diffusion lengths over the entire AFSD cycle for 82 J/mm$^2$ and 116 J/mm$^2$ conditions.

The above equation was solved numerically to obtain the diffusion length of Al during heating and cooling events of each thermal cycle experienced by a location in layer 3. Figure 6 provides cumulative diffusion length with each thermal cycle in 82 J/mm$^2$ and 116 J/mm$^2$ samples. The total diffusion spread of Al atoms in 116 J/mm$^2$ sample is broader (14 μm) compared to 82 J/mm$^2$ (4 μm) due to comparatively lower linear deposition speed and higher heat accumulation for the 116 J/mm$^2$ sample. Such broad diffusion lengths of Al atoms can effectively dissolve the precipitate and homogenize the alloy during the thermal process such as AFSD. During the cooling phase, the diffusion of Al in Mg decelerated and became sluggish making it difficult for the $\beta$ phase to re-precipitate. Therefore, the AFSD samples had a significant reduction in $\beta$ phase fraction (Fig. 4b–c). Such a thermokinetics driven microstructure evolution affected the mechanical response of the AFSD samples.

**Figure 7:**
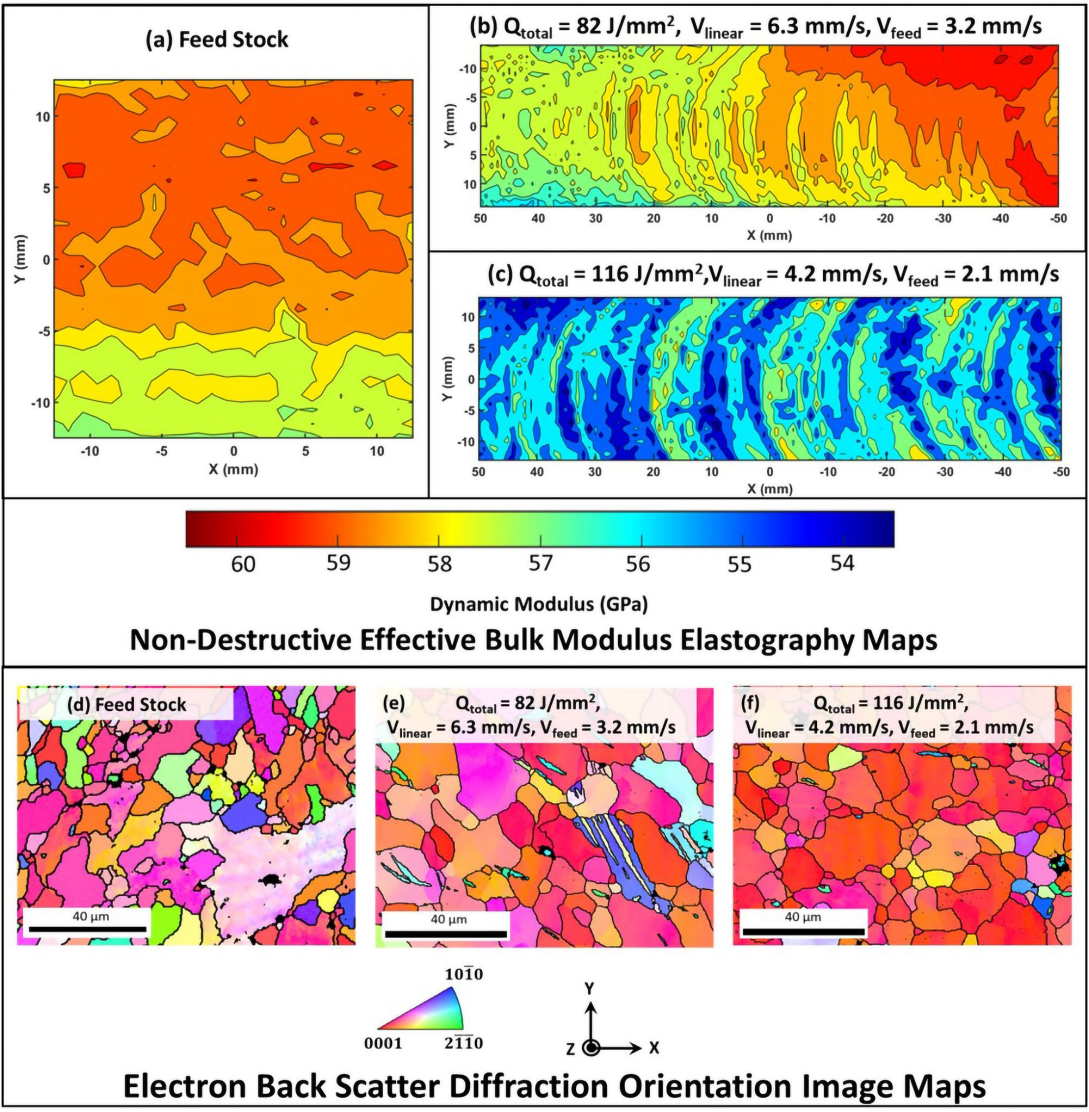
Bulk modulus data obtained by EBME technique corresponding to (**a**) feed stock, (**b**) 82 J/mm$^2$, and (**c**) 116 J/mm$^2$ samples along with high magnification OIM data for (**d**) feed stock, (**e**) 82 J/mm$^2$, and (**f**) 116 J/mm$^2$ samples.

The AFSD samples were first examined using non destructive EBME technique described in the “Methods and materials” Section. The scanned data of the three-dimensional volume of the AFSD samples from the top XY plane was collected and rendered as contour plots of the average spatial distribution of dynamic bulk modulus. Along the same lines, the contour plot of dynamic bulk modulus was rendered for the feed stock scanned from the normal plane. These contour plots of dynamic bulk modulus are presented in Fig. 7a, b, and c, corresponding to feed stock, 82 J/mm$^2$, and 116 J/mm$^2$, respectively. The spatial distribution of dynamic bulk modulus for the feed stock was confined to the narrow range of 57.5–60.0 GPa (Fig. 7a). A similar range of dynamic bulk modulus (57.0–60.5 GPa) was recorded for the 82 J/mm$^2$ AFSD sample (Fig. 7b). However, this range was considerably shifted towards lower modulus values of 54.5–57.0 GPa for the 116 J/mm^2^ AFSD sample (Fig. 7c). The values of dynamic bulk modulus obtained via ultrasound qualitatively reflect the extent of residual stress in the material^43,44^. The elastic modulus is the inherent property of the material associated with inter-atomic potential energy and spacing. The presence of residual stress is associated with the elastically strained lattice, which affects the inter-atomic spacing and decreases the potential energy, thereby reducing the elastic modulus of the material.

The feed stock is likely to have the lowest residual stresses as it received H24 treatment^38^, which justifies a higher dynamic modulus of feed stock. However, the difference in the dynamic moduli of AFSD samples indicates a difference in residual stress. This discrepancy can be addressed by analyzing the OIM micrographs at higher magnification (Fig. 7d–f). The OIM micrographs taken at higher magnification indicated the presence of mechanical twins in both AFSD samples, while they were not observed in the feed stock. Moreover, it can be observed that mechanical twins were more prevalent in 82 J/mm$^2$ sample while they were scarcely observed in 116 J/mm$^2$ sample (Fig. 7e and f). The presence of mechanical twins in the 82 J/mm$^2$ sample indicates that deformation is heavily accompanied by twins in addition to slip. Moreover, the formation of mechanical twins accommodates extensive lattice strain^59,60^, thereby reducing the overall residual stress in 82 J/mm$^2$ sample, which is also reflected in its EBME map with a dynamic modulus similar to feed stock (Fig. 7a and b). On the contrary, the scarcity of mechanical twins in 116 J/mm$^2$ sample suggests deformation majorly via slip. Moreover, the strain rate generated during the 116 J/mm$^2$ sample fabrication due to lower linear velocity is likely to be lower^37^. Also, the longer duration of thermal cycles associated with the fabrication of 116 J/mm$^2$ sample sustains the heat for a longer duration (Fig. 5b). These rationalize the scarcity of mechanical twins in 116 J/mm$^2$ sample. As the slip accommodates lower lattice strain, the residual stress in 116 J/mm$^2$ sample is likely to be higher compared to 82 J/mm$^2$ sample, which is justified through EBME maps showing reduced dynamic modulus (Fig. 7b and c).

**Figure 8:**
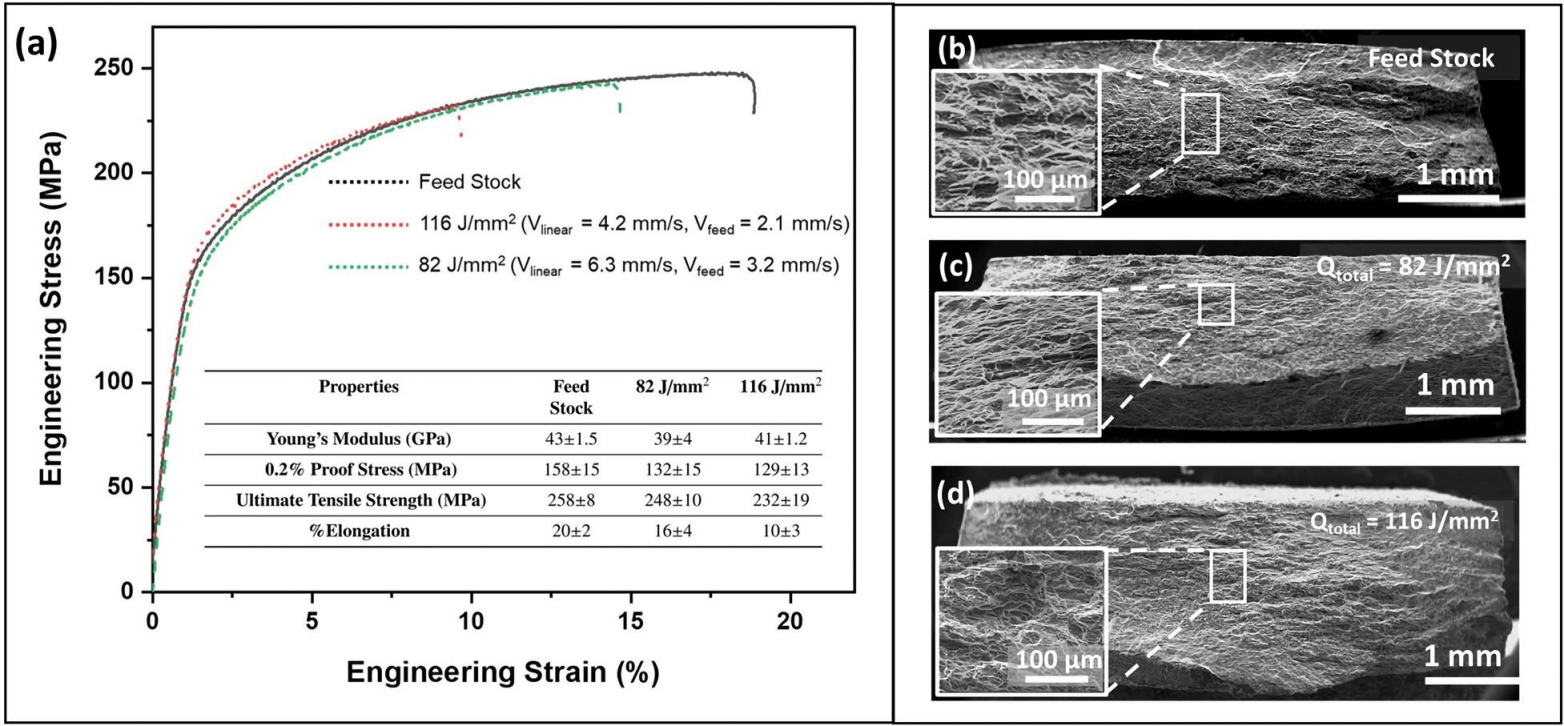
(**a**) Representative stress strain curves along with tabulated mechanical properties for feed stock as well as AFSD samples and fractographs corresponding to (**b**) feed stock, (**b**) 82 J/mm$^2$, and (**c**) 116 J/mm$^2$ samples. The insets in the fractographs present high magnification views of the corresponding highlighted regions.

Engineering stress stain curves for AZ31B-Mg feed stock and AFSD samples possessed nearly identical slope in the elastic regime indicating similar Young’s modulus of 40 GPa for these samples (Fig. 8). However, there was a reduction of $\sim$ 30 MPa in the 0.2% PS for the AFSD samples compared to the feed material at 158 ± 15 MPa (Fig. 8). Such a reduction could be attributed to an increase in the average grain size by 4–7 μm (Fig. 3) and the reduction in fraction of Mg$_{17}$Al$_{12}$ precipitates in the AFSD samples compared to the feed stock (Fig. 4). These two effects simultaneously led to reduction in the barriers for dislocation motion, thus lowering the 0.2% PS for the AFSD samples compared to the feed stock. The UTS of feed stock was 258 ± 8 MPa which was marginally higher by 10 MPa and 26 MPa compared to 82 J/mm$^2$ and 116 J/mm$^2$ AFSD samples respectively (Fig. 8). The AZ31B-Mg feed stock material elongation was 20 ± 2%. On the other hand, the AFSD samples exhibited lower elongation of 16 ± 4% and 10 ± 4% for 82 J/mm$^2$ and 116 J/mm$^2$ samples respectively (Fig. 8). Such a reduction in elongation could be attributed to evolution of strong basal texture on the XY surface/subsurface of the AFSD samples (Fig. 3). The samples were loaded in Y direction (perpendicular to the build direction)(Fig. 1c). During uni-axial tensile loading, the lattice rotates in such a way that the basal slip plane normal is tilted towards loading axis^61^. The material accommodates deformation until the basal plane normal becomes perpendicular to the loading axis at which the Schmid factor of the slip planes approaches zero. In the current case, the base material was associated with a diffused basal texture with 15° offset from the 0001 basal pole (Fig. 3a). On the other hand the 82 J/mm$^2$ sample possessed a sharp basal texture with 35° offset (Fig. 3b). Such an offset with sharper texture requires higher amount of deformation for bringing the basal plane normal perpendicular to the loading axis. As a result, the 82 J/mm$^2$ sample experienced a higher elongation among the AFSD samples. On the other hand, although the basal texture was sharp, the offset was lower for the 116 J/mm$^2$ sample (Fig. 3c), hence accommodating lesser deformation than the 82 J/mm$^2$ sample, before the basal plane normal was aligned perpendicular to the loading axis, resulting in lower elongation. As a note, reduction in mechanical properties for AFSD fabricated Mg alloys has been reported before ^22,23^. However, these works lacked the explanation about the correlation of process thermokinetics driven multi scale microstructure evolution with the resultant mechanical behavior.

As a next step in analysis, the fracture surfaces of the broken samples from the tensile tests were observed using secondary electron mode SEM (Fig. 8b–d). The fracture surfaces revealed a quasi brittle failure mode with cleavage like fracture for both feed stock and AFSD AZ31B Mg samples. It has been reported that Mg based materials inherently have a low fracture toughness and usually exhibit a cleavage fracture mode during quasi-static tensile loading in a vast temperature range^62–64^.

## Conclusions

Current work explored solid state additive manufacturing of AZ31B-Mg alloy via AFSD process. The average Archimedes density values of AFSD fabricated samples were $1.761 \pm 0.006$ and $1.768 \pm 0.006$ g/cm$^3$ for the processing conditions corresponding to the input energies of 82 and 116 J/mm$^2$ respectively compared to the Archimedes density value of 1.77 g/cm$^3$ for the feed stock material. This translates into relative density values of 99.4 and 99.8% for 82 and 116 J/mm$^2$ samples respectively indicating a reasonable consolidation of the AFSD fabricated AZ31B Mg material. The temporal and spatial variation of temperature during AFSD process was predicted using a multi layer computational process model. The temperature experienced by the material during the deposition and due to subsequent reheating as a result of added layers on the top remained in single $\alpha$-Mg phase field region (above 200 °C). Such distinct thermokinetic conditions led to an average grain size of 15 ± 4 and 18 ± 3 μm for 82 J/mm$^2$ and 116 J/mm$^2$ AFSD conditions respectively compared to 11 ± 3 μm for the feed stock. The AFSD processed samples developed a strong basal texture on the top surface. The feed stock exhibited a diffused texture aligned 15° offset to 0001 pole. Both AFSD samples possessed a strong basal texture on the top surface aligned 35 and 15° offset to 0001 pole for 82 J/mm$^2$ and 116 J/mm$^2$ conditions respectively. The higher temperatures experienced by the AFSD material (greater than 200 °C) during deposition followed by cooling down to room temperature with 1–2 °C/s rates resulted in a marked reduction in fraction of nano scale $\beta$ phase in the AFSD samples compared to the feed stock material. AFSD sample deposited with 82 J/mm$^2$ revealed a higher amount of twinning compared to 116 J/mm$^2$ and feed stock material. As a result, the non destructively evaluated bulk modulus was lower for 116 J/mm$^2$ sample (54.5-57.0 GPa) compared to the 82 J/mm$^2$ sample (57.0-60.5 GPa) and feed stock (57.5-60.0 GPa). Feed stock and AFSD AZ31B-Mg samples exhibited nearly same Young’s modulus of $\sim$ 40 GPa during uni-axial tensile tests. However, AFSD sample deposited with 82 and 116 J/mm$^2$ input energies possessed a 0.2%PS of 132 ± 15 MPa and 129 ± 13 respectively which was lower than 0.2% PS of 158 ± 15 for the feed stock. UTS of AFSD samples was 248 ± 10 and 232 ± 19 MPa for 82 and 116 J/mm$^2$ conditions respectively. The feed stock UTS was 258 ± 8 MPa. The elongation of the AFSD AZ31B-Mg was lower by 4 % and 10 % for 82 and 116 J/mm$^2$ process conditions respectively compared to the feed stock at 20%. The distinct thermokinetic effects involving multiple reheating cycles during AFSD led to the unique microstructure having a coarser grain size and reduced fraction of $\beta$ phase leading to such a reduction in tensile properties for the AFSD AZ31B-Mg compared to the feed stock.

## Data Availability

The data sets used and/or analyzed during the current study will be made available from the corresponding author on a reasonable request.
